# Improving the Adhesion of Multi-Walled Carbon Nanotubes to Titanium by Irradiating the Interface with He^+^ Ions: Atomic Force Microscopy and X-ray Photoelectron Spectroscopy Study

**DOI:** 10.3390/nano14080699

**Published:** 2024-04-17

**Authors:** Petr M. Korusenko, Egor V. Knyazev, Olga V. Petrova, Denis V. Sokolov, Sergey N. Povoroznyuk, Konstantin E. Ivlev, Ksenia A. Bakina, Vyacheslav A. Gaas, Alexander S. Vinogradov

**Affiliations:** 1 Department of Solid State Electronics, V.A. Fock Institute of Physics, St. Petersburg State University, 7/9 Universitetskaya Nab., 199034 Saint Petersburg, Russia; 2 Department of Physics, Omsk State Technical University, 11 Mira Prosp., 644050 Omsk, Russia; 3 Laboratory of Physics of Nanomaterials for Chemical Current Sources, Omsk Scientific Center of SB RAS, 15 Karl Marx Prosp., 644024 Omsk, Russia; 4 Institute of Physics and Mathematics, Komi Science Centre, Ural Branch of the Russian Academy of Sciences, 167982 Syktyvkar, Russia

**Keywords:** multi-walled carbon nanotubes (MWCNTs), irradiation with helium ions, interfacial adhesion, atomic force microscopy (AFM), X-ray photoelectron spectroscopy (XPS), oxygen-containing functional groups (OCFGs)

## Abstract

A complex study of the adhesion of multi-walled carbon nanotubes to a titanium surface, depending on the modes of irradiation with He^+^ ions of the “MWCNT/Ti” system, was conducted using atomic force microscopy and X-ray photoelectron spectroscopy. A quantitative assessment of the adhesion force at the interface, performed using atomic force microscopy, demonstrated its significant increase as a result of treatment of the “MWCNT/Ti” system with a beam of helium ions. The nature of the chemical bonding between multi-walled carbon nanotubes and the surface of the titanium substrate, which causes this increase in the adhesion of nanotubes to titanium as a result of ion irradiation, was investigated by X-ray photoelectron spectroscopy. It was established that this bonding is the result of the formation of chemical C–O–Ti bonds between titanium and carbon atoms with the participation of oxygen atoms of oxygen-containing functional groups, which are localized on defects in the nanotube walls formed during ion irradiation. It is significant that there are no signs of direct bonding between titanium and carbon atoms.

## 1. Introduction

With the ongoing miniaturization of electronic devices, the industry of portable chemical energy sources such as lithium-ion batteries and supercapacitors is developing rapidly [1,2]. The use of nanocomposite materials based on multi-walled carbon nanotubes (MWCNTs) and conductive polymers as supercapacitors’ electrode materials is considered a way to improve the energy characteristics of these devices. The improvement is due to a combination of two factors. Firstly, there is the high specific surface area of MWCNTs, which increases the electrical double layer capacity, and secondly, a conductive polymer contributes to the capacity through redox reactions (pseudo-capacity) [3,4]. However, the practical application of such a material is significantly hampered by the poor adhesion of MWCNTs to the conductive substrate.

Currently, researchers are trying to solve this problem by adding polymer binders during the preparation of the electrode material, such as polyvinylidene fluoride (PVDF), carboxymethylcellulose (CMC), styrene-butadiene latex (SBL), etc. [5,6,7]. However, this approach is accompanied by a significant decrease in the conductivity of the electrode material, which creates a need to develop new binder compositions and other methods for increasing the adhesion of the electrode to the substrate.

As a promising alternative approach for improving the adhesion between the carbon layer and the substrate, the method of irradiation with Ar^+^ ions has been tested using the MWCNT/Ti interface as an example [8]. However, this study focused on a qualitative characterization of the interfacial adhesion, without quantification, which makes it difficult to understand the nature of the increased adhesive strength between MWCNTs and titanium.

According to [9,10,11,12,13,14], the adhesive strength of a carbon nanotube layer on a substrate can be measured using macro- and micromechanical techniques. For these materials, adhesion strength is determined by the applied force (tensile, shear, or peel) required to break the bond between the two materials. However, qualitative methods for evaluating adhesion in these systems are not reliable, as they lack a standard evaluation procedure [15,16,17].

In [9,14,18,19,20], the prospects for using atomic force microscopy (AFM) to characterize the interaction between carbon nanostructures and a substrate were investigated. These studies demonstrated that the AFM technique can be used to quantify the strength of this interaction, which opens up the possibility of numerical comparison and control of this strength for different carbon-nanostructure–substrate combinations.

The purpose of this work was to conduct a complex experimental investigation of the initial and irradiated MWCNT/Ti systems using atomic force microscopy (AFM) and X-ray photoelectron spectroscopy (XPS). In addition, scanning electron microscopy (SEM) and energy-dispersive X-ray spectroscopy (EDS) were used to characterize the morphology and the elemental composition of the samples, respectively. The main tasks of this research were to quantify the adhesion strength between MWCNTs and the titanium surface after various irradiation parameters using AFM, as well as to clarify the nature of the increased adhesion strength induced by ion irradiation using XPS.

## 2. Materials and Methods

Commercial MWCNTs (MWCNTs-2, produced by the G.K. Boreskov Institute of Catalysis, Siberian Branch of the Russian Academy of Sciences (SB RAS)) [21] with a specific surface area of 250 m^2^/g and an average outer diameter of 4 to 21 nm were used in this study. To deposit the MWCNT layer on the titanium surface, firstly, a suspension was prepared by dispersing the nanotubes in isopropyl alcohol. For this purpose, 5 mg of MWCNT powder was treated in 15 mL of alcohol in a Jeken Ultrasonic Cleaner (model PS-06A, Dongguan City, China) for 5 h. The MWCNT layer was deposited on the titanium substrate (~50 μm thick foil), which was heated to 70 °C, by spraying the suspension using an air compressor with 10 W power and a 10 L/min flow rate. The thickness of the prepared MWCNT layers was ~1 μm.

Ion beam treatment of the surface of the MWCNT/Ti system with He^+^ ions with an average energy of 20 keV was carried out for 10, 20 and 30 min in an implanter at the Omsk Scientific Center of the Siberian Branch of the Russian Academy of Sciences. The ion beam was generated by ionizing helium atoms in a Penning discharge under the influence of crossed magnetic and electric fields. The average energy of the ion beam was chosen based on the expected thickness of the MWCNT layer on Ti, which was calculated from an analysis of the projective range of helium ions through the MWCNT layer using the SRIM-2013 program [22]. The beam fluence and the degree of defectiveness of the MWCNT layer on the substrate varied depending on the duration of exposure.

The morphology and elemental composition of the “MWCNT/Ti” system were studied before and after irradiation via the SEM and EDS methods, using a JEOL JSM-6610LV (Tokyo, Japan) electron microscope equipped with an Inca-XAct energy-dispersive spectrometer.

Additionally, the morphology of the samples was also characterized using AFM in the semi-contact mode (with a cantilever spring constant of 17 N/m). In contrast, the contact mode (with a cantilever spring constant of 34 N/m) was used to quantify the adhesion strength of the MWCNT layer to the titanium substrate and its changes after ion irradiation. The surface of the MWCNT layer was scanned at 50 µm/s and a loading force, the probe to sample in the range of 0.4–26 μN, which increased by 0.2 μN with each subsequent scan. The loading force (*F*, µN) was determined from the signal of the laser-optical cantilever system (*DFL*, V) to be *F* = *k* · ∆*z* · *DFL*, where *k* is the spring constant of the cantilever, and ∆*z* is the angle of the experimental force–distance curve, nm/V. All the measurements were carried out using the MFP-3D atomic force microscope (Asylum Research, Santa Barbara, CA, USA) with HA_HR cantilevers (TipsNano, Tallinn, Estonia). The adhesion of the layer to the substrate was determined based on the magnitude of force required to separate the layer from the substrate while gradually increasing the force applied by the probe to the MWCNT surface.

Clarification of the nature of increased adhesion of MWCNTs to due to ion irradiation of MWCNT/Ti and detailed characterization of the mechanisms of this phenomenon were conducted based on the results of an analysis of the shapes and binding energies of the C 1s, O 1s, and Ti 2p core-level photoelectron (CL PE) spectra for the initial and irradiated “MWCNT-Ti” systems. Measured PE spectra were also used to obtain additional information about the elemental compositions of these systems. All measurements were performed using an ESCALAB 250Xi laboratory electron spectrometer (Thermo Fisher Scientific, Waltham, MA, USA) with monochromatized AlKα radiation (*hν* = 1486.6 eV) at the St. Petersburg State University Research Park (Centre for Physical Methods of Surface Investigation). Survey and CL (C 1s, O 1s, and Ti 2p) PE spectra were measured with analyzer pass energies of 50 eV and 20 eV, respectively. Calibration of the binding energy scale was accomplished using a pure gold foil, for which the position of the Au 4f_7/2_ PE line and Fermi level were measured. A detailed analysis of the CL PE spectra was performed by approximating the spectra with a set of individual components using the Casa XPS 2.3.16 software [23].

## 3. Results and Discussion

### 3.1. SEM

**Figure 1:**
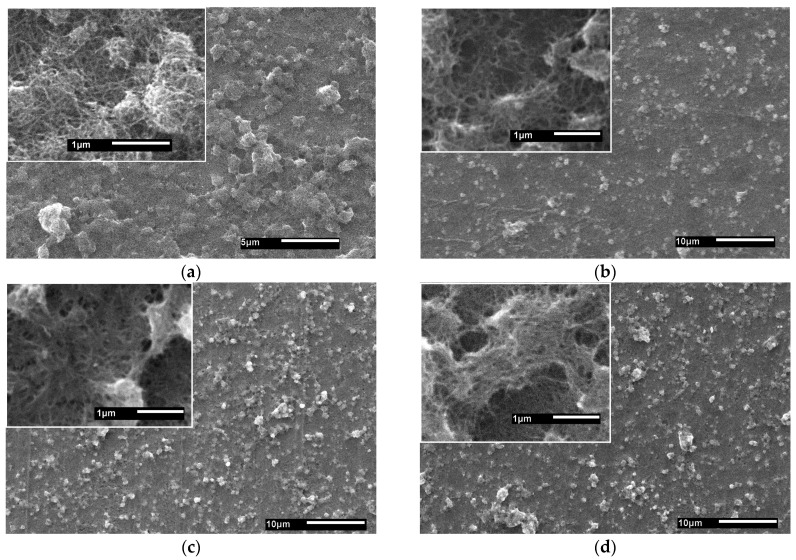
SEM images of “MWCNT/Ti” surface layer: (**a**) initial and irradiated with He^+^ for (**b**) 10 min, (**c**) 20 min, and (**d**) 30 min.

The SEM data (Figure 1a) show the presence of a uniform continuous layer of MWCNTs on the titanium substrate, with an average outer diameter of the nanotubes of up to 20 nm. The nanotubes in this layer are mainly oriented parallel to the substrate and form numerous intersections and overlaps with each other. On the surface of the MWCNT layer, individual nanotube agglomerates of up to 1 μm in size without clearly defined boundaries are observed, which are produced during the layer deposition.

Irradiation of the MWCNT layer on a Ti substrate with a helium-ion beam was carried out at different exposure times. As a result of 10 min irradiation, a clearer boundary of agglomerates can be observed on the surface of the layer due to rounded cavities (Figure 1b). The formation of these cavities is probably associated with the partial destruction of nanotubes by helium ions. With increasing irradiation time, the number and size of cavities between the nanotubes increase to approximately 500 nm (Figure 1c,d). In the magnified SEM images of the MWCNT layer (inset in Figure 1c,d), there are signs of welding between the individual MWCNTs. Thus, as a result of irradiation with a helium-ion beam, pronounced morphological changes in the surface layer of MWCNTs are observed.

### 3.2. EDS

The average elemental analysis data obtained through the EDS technique are shown in Table 1. As the time of ion beam treatment of the MWCNT surface increases, there is an increase in the titanium concentration and a decrease in the carbon concentration in the examined regions of samples. This result can be explained by a decrease in the thickness and density of the MWCNT layer during irradiation with helium ions.

### 3.3. AFM

**Figure 2:**
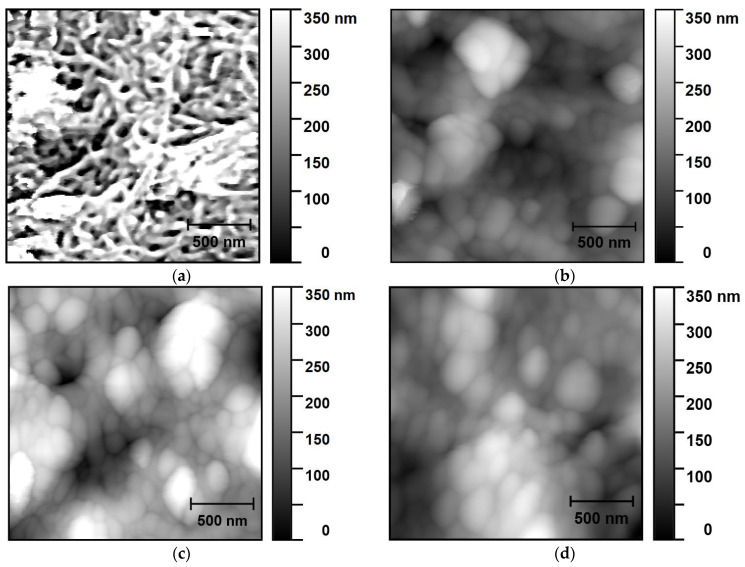
AFM images of “MWCNT/Ti” surface layer: (**a**) initial and irradiated with He^+^ for (**b**) 10 min, (**c**) 20 min and (**d**) 30 min.

Figure 2 shows AFM images of the surface layer of a “MWCNT/Ti” system obtained in the semi-contact mode before and after irradiation. On the surface of the untreated sample (Figure 2a), there is a network of intersecting thread-like structures of MWCNTs, which are also visible on the SEM image (Figure 1a).

After the irradiation, the thread-like structures disappear, which may be due to the compacting of the surface layer as a result of the welding of nanotubes. In the images of the irradiated layers, cavities between nanotubes can be seen (Figure 2b–d), which agrees with the SEM data (Figure 1).

Quantitative evaluation of the interfacial adhesion between the surface MWCNT layer and the Ti substrate was carried out using the AFM contact mode technique. The destruction of the MWCNT layer on the Ti substrate was assessed by scanning the sample in the semi-contact mode. The adhesion force was determined as the loading force applied by the AFM probe, at which a layer of nanotubes was removed from the substrate surface in the scanning area. The results of the average adhesion forces for the MWCNT layer before and after irradiation with helium ions are presented in Table 2.

As can be seen in Table 2, the adhesion strength between MWCNTs and the Ti substrate depends on the time of ion beam treatment. Ion irradiation with helium for 10 min does not affect the adhesion strength, whereas an increase in adhesion is observed after 20 and 30 min. It is important to note that the duration of ion exposure generally affects the concentration of defects formed in the “MWCNT/Ti” system during its irradiation with helium ions. Consequently, an increase in the ion treatment time contributes to an increase in the concentration of point and extended structural defects at the “MWCNT/Ti” interface. It seems that this has a positive effect on the adhesive strength of the MWCNT layer to the titanium substrate. In addition, oxygen-containing functional groups (OCFGs) can also attach to the defective surface of carbon nanotubes from the residual gas environment of the implanter’s working chamber [24]. This can also affect the nature of the bonding between the MWCNT layer and the titanium substrate.

### 3.4. XPS 

**Figure 3:**
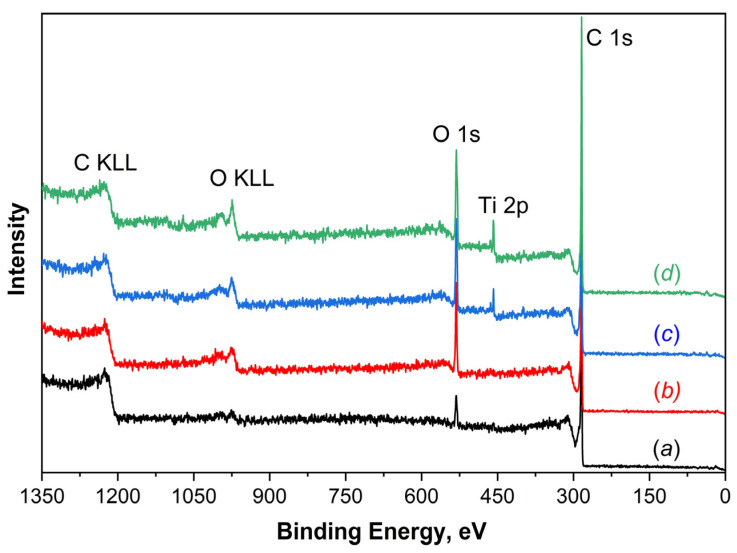
Survey PE spectra of “MWCNT/Ti” surface: (*a*) initial and irradiated with He^+^ for (*b*) 10 min, (*c*) 20 min, and (*d*) 30 min.

Elemental analysis of untreated and irradiated samples was also carried out by XPS by analyzing the intensities of individual PE lines of elements in the survey PE spectra (Figure 3). Quantitative determination of element concentrations was performed by the method of elemental sensitivity coefficients. The results are summarized in Table 3. The general trend in the changes in the percentage of individual elements observed in the XPS spectra corresponds to the changes in the EDX data (see Table 2). However, when comparing the results from these two methods, it should be taken into account that the XPS is a surface-sensitive method and probes the sample to a depth of approximately 3–5 nm, whereas the EDX method examines a surface layer 1.2–1.5 µm thick. Therefore, EDX data mainly contain information from the titanium substrate, resulting in a significantly higher concentration of titanium atoms and a lower concentration of carbon and oxygen compared to the surface-sensitive XPS method.

According to elemental analysis using XPS, it was found that as the irradiation time of the MWCNT layer on a titanium substrate rises, the concentration of oxygen and titanium atoms in the surface layers increases, while the concentration of carbon atoms simultaneously decreases (Table 3). This change in concentration is associated with a reduction in the thickness and density of the nanotube layer, as well as the appearance of cavities during ion irradiation. The increase in oxygen percentage is likely due to the formation of defects in the MWCNT structure and the attachment of OCFGs to them, as well as an increasing contribution from the oxide layer on the titanium substrate surface [8].

A detailed analysis of the C 1s, O 1s, and Ti 2p PE spectra of MWCNT/Ti samples measured before and after irradiation with a helium-ion beam allowed us to gain the most complete information about the nature of the increased interfacial adhesion in these systems caused by irradiation.

**Figure 4:**
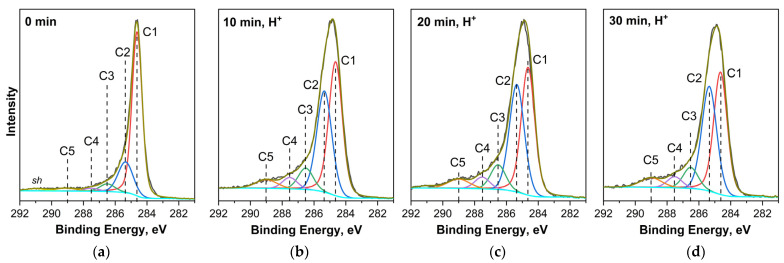
C 1s PE spectra of “MWCNT/Ti” surface: (**a**) initial and irradiated with He^+^ for (**b**) 10 min, (**c**) 20 min, and (**d**) 30 min.

Figure 4 shows the C 1s PE spectra of “MWCNT/Ti” before and after ion treatment. The spectrum of the sample before irradiation (Figure 4a) contains five components that correspond to graphite-like sp^2^ carbon C=C (~284.6 eV, C1), sp^3^ carbon C–C in diamond and/or carbon located near oxygen-containing functional groups [C*–C(O)] (~285.5 eV, C2), carbon in the C–O (~286.4 eV, C3) and C=O (~287.5 eV, C4) groups, as well as carbon in the COOH– groups (~289 eV, C5) [24,25,26]. The high-energy maximum *sh* (~291 eV) represents a satellite, which is typical for C 1s PE spectra of sp^2^ carbon atoms in systems with a high degree of graphitization and is associated with the π→π* shake-up process occurring simultaneously with C 1s photoionization [25].

Figure 4 clearly shows that as a result of irradiation, significant changes occur in the shape of the C 1s PE spectrum, namely, an increase in its full width at half maximum (FWHM) from 0.7 eV to 1.45 eV, a redistribution of the relative intensities of its components, and the disappearance of the shake-up satellite. All this indicates a change in the content of carbon atoms in different chemical states, the possible formation of additional chemical bonds between the carbon atoms of MWCNTs and other atoms of the system, as well as significant defect formation in the graphite-like walls of nanotubes. A detailed comparison of these spectra (Figure 4b–d) reveals that the change in FWHM for the C 1s PE spectra of the irradiated MWCNT/Ti system is due to a redistribution of the relative intensities of the C1 component (sp^2^ carbon) and the C2, C3, and C4 components, which belong to the carbon atoms interacting with oxygen atoms. These findings in the C 1s PE spectra of irradiated samples indicate that the outer walls of MWCNTs undergo significant structural damage as a result of ion irradiation, which leads to an increase in the degree of wall imperfection and the anchorage of OCFGs at the sites of formed defects. Here, an increase in the ion beam irradiation time from 10 to 30 min is accompanied by an intensification of the formation of structural defects and oxidation of the outer walls of MWCNTs. It is important to note that in all C 1s PE spectra of the irradiated samples, there is no component at a binding energy of ~282.1 eV, corresponding to C 1s electrons in titanium carbide [27].

**Figure 5:**
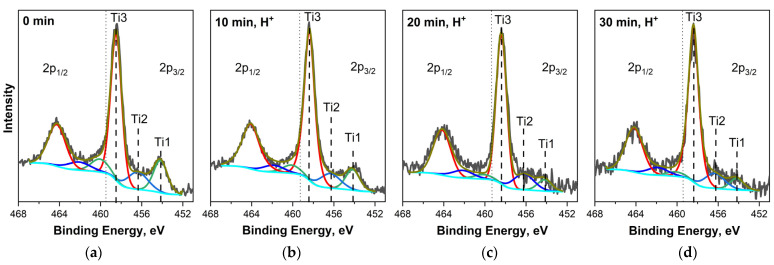
Ti 2p_1/2,3/2_ PE spectra of “MWCNT/Ti” surface: (**a**) initial and irradiated with He^+^ for (**b**) 10 min, (**c**) 20 min, and (**d**) 30 min.

Figure 5 shows the Ti 2p_1/2,3/2_ PE spectra of the MWCNT/Ti system measured before and after ion treatment. In the following, we will only consider the most intense Ti 2p_3/2_ spectrum. In this spectrum of the “MWCNT/Ti” system before and after irradiation, three components are observed, which correspond to metallic titanium atoms (~454.2 eV, Ti1), partially oxidized titanium atoms in TiO_1−x_ and TiO_2−x_ (~456.4 eV, Ti2), as well as titanium atoms in TiO_2_ (~458.4 eV, Ti3) [28,29,30]. It should be noted that the PE lines of TiO_1−x_ and TiO_2−x_ oxides in our previous study [8] were considered separate components, but due to difficulties of approximation, in this work, they were combined into one component.

From these data, it is clear that on the surface of the titanium substrate in all samples, oxide compounds of titanium atoms with varying degrees of oxidation dominate, while maintaining a certain proportion of Ti^0^ atoms. Moreover, in the spectra of the samples under consideration, as the irradiation time increases, a decrease in the relative intensity of the “metallic” component Ti1 and an increase in the intensity of the “dioxide” component Ti3 are observed. Such changes may be associated with additional oxidation of titanium atoms during ion beam treatment. It should be emphasized that the Ti 2p_3/2_ and C 1s PE spectra of the irradiated samples do not contain components at binding energies of ~455.4 eV and ~282.1 eV, associated, respectively, with Ti and C atoms in titanium carbides [27]. This observation suggests that direct chemical bonding between carbon and titanium atoms is absent from the studied MWCNT/Ti systems. Thus, we can conclude that the observed increase in adhesion upon ion irradiation of “MWCNT/Ti” cannot occur due to direct bonds between the atoms of the titanium substrate and carbon atoms in the MWCNT layer.

**Figure 6:**
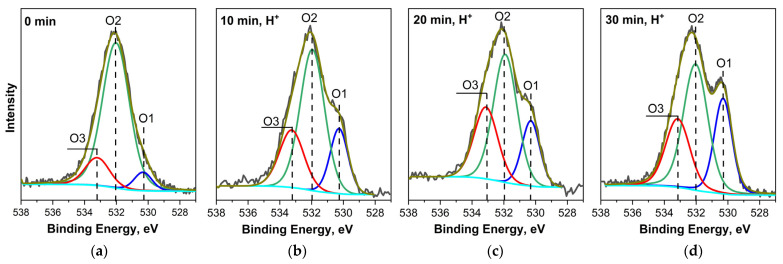
O 1s PE spectra of “MWCNT/Ti” surface: (**a**) initial and irradiated with He^+^ for (**b**) 10 min, (**c**) 20 min, and (**d**) 30 min.

In the O 1s PE spectrum of the untreated MWCNT/Ti system (Figure 6a), three components can be distinguished, which correspond to oxygen atoms in TiO_2_ (~530.3 eV, O1), as well as in OCFGs with double and single chemical bonds between the atoms carbon and oxygen: C=O (~532 eV, O2) and C–O (~533.1 eV, O3) [28,29].

Irradiation of MWCNTs on a titanium substrate with helium ions for 10 min leads to a change in shape and broadening of the O 1s PE spectrum. Both effects are due to an increase in the intensities of the O1 and O3 components, which corresponds to oxygen in titanium oxides and in carbon compounds with a single chemical C–O bond. After 20 min of treatment of “MWCNT/Ti” with an ion beam, a further increase in the relative intensities of the O1 and O3 components is observed. At the same time, when this system is irradiated for 30 min, an increase in the relative intensity is detected only for the O1 component, while the intensity of the O3 component, on the contrary, decreases. The results of the three-component approximation of O 1s PE spectra for the MWCNT/Ti systems before and after irradiation are presented in Table 4.

The discovered change in the relative O1 intensity may be partly due to a decrease in the thickness of the MWCNTs layer as a result of its partial destruction by a helium-ion beam and an increase in the contribution of the titanium substrate surface to the spectrum. A possible reason for the nonmonotonic change in the relative intensity of O3 may be the ion-stimulated formation and destruction of C–O groups localized on the surface of carbon nanotubes on structural defects, the number of which increases with increasing irradiation time. A change in the amount of these C–O groups was previously observed in the C 1s PE spectra of irradiated samples (Figure 4).

It is now known that during the preparation of composites based on carbon nanomaterials (graphene or MWCNTs), chemical adhesion occurs between the carbon nanomaterial and metal oxide particles TiO_x_, SnO_x_, or CuO deposited on its surface [26,31,32,33,34,35,36]. This phenomenon is caused by the formation of direct chemical C–M bonds [34,35,36] or C–O–M bonds as a result of the linking of metal atoms M to C–O groups that appear on the surface of carbon materials due to ion bombardment in an oxygen-containing atmosphere [8,26]. In our case, a combined analysis of the C 1s and Ti 2p PE spectra showed that direct C–Ti bonds are not formed during ion irradiation. Therefore, the formation of C–O–Ti chemical bonds is more likely. At the same time, the energy position of the component corresponding to these bonds in the O 1s PE spectrum is close to the position of the O2 component corresponding to the O 1s PE line of C=O groups on the surface of the carbon material, which significantly complicates the interpretation of experimental spectra [31]. Therefore, we used information on the contents of C=O groups, obtained by taking into account the proportion of the O2 component relative to the total oxygen content for a specific sample (Table 4). When comparing these data, it is clear that the concentration of the O2 component barely changes with the duration of ion treatment, which occurs since, it seems, the proportion of carbon atoms in C=O bonds also increases. In view of this, it is more logical to consider the change in the O3 component, which is associated with C–O groups. It can be clearly seen (Table 4) that its concentration changes significantly when moving from the untreated MWCNT/Ti system to the irradiated ones. In this case, the maximum content of these groups is detected after irradiation with an ion beam for 20 or 30 min, which correlates with the change in the interfacial adhesion values of the MWCNT/Ti system obtained from AFM data (Table 2). These results allow us to conclude that when “MWCNT/Ti” systems are irradiated with helium ions, C–O–Ti chemical bonds are formed at the interface, which lead to an increase in the adhesion of MWCNTs to Ti. It should be emphasized that the formation of these bonds occurs with the participation of C–O functional groups, which are formed in an oxygen-containing atmosphere of an implanter during ion bombardment on the surface of MWCNTs and are localized to its structural defects.

## 4. Conclusions

A detailed study of MWCNT layers deposited on a titanium substrate has shown significant changes in the morphology and chemical composition of these samples as a result of their treatment with helium ions. It has been established that He^+^ ion irradiation of the MWCNT/Ti system leads to welding of individual nanotubes, their partial destruction with the formation of cavities, and, as a consequence, a decrease in the density of the MWCNT layer, which is confirmed by SEM, EDX, AFM, and XPS data. Meanwhile, increasing the ion beam irradiation time from 10 to 30 min intensifies these processes.

A quantitative assessment of the interfacial adhesion force of MWCNTs to Ti as a function of the time of exposure to an ion beam was carried out. It was shown that the highest value of the interfacial adhesion force, amounting to 18.3 μN (which is 57% higher than in the case of an untreated sample), is achieved when the MWCNT/Ti system is irradiated for 20 min. This result is explained by the formation of C–O–Ti bonds as a result of the chemical bonding of titanium atoms and oxygen-containing functional groups on the surface of MWCNTs, which are formed in the residual oxygen-containing atmosphere of the ion implanter near point and extended structural defects during irradiation with the ion beam. It is important that, in this case, the formation of direct Ti–C bonds between titanium and carbon atoms does not occur.

The results obtained will allow us to approach the problem of improving interfacial adhesion at the “MWCNT–current-collecting substrate” interface without using a binder, which will ensure the development of new highly efficient electrode materials based on carbon nanotubes and electrically conductive polymers for electrochemical applications (lithium-ion batteries and supercapacitors).

## Figures and Tables

**Table 1:** Elemental compositions of initial and He^+^-irradiated “MWCNT/Ti” systems according to EDS data.

Sample	Concentration, at. %		
	[C]	[O]	[Ti]
Initial MWCNT/Ti	40.8	5.3	54.0
MWCNT/Ti, irradiated for 10 min	35.7	4.2	60.1
MWCNT/Ti, irradiated for 20 min	29.7	7.4	62.9
MWCNT/Ti, irradiated for 30 min	25.7	5.4	68.9

**Table 2:** Values of adhesion strength between MWCNTs and titanium substrate for initial and He^+^-irradiated “MWCNT/Ti” systems for 10, 20, and 30 min.

	Sample	Initial MWCNT/Ti	MWCNT/Ti, Irradiated for 10 min	MWCNT/Ti, Irradiated for 20 min	MWCNT/Ti, Irradiated for 30 min
Adhesion Strength					
*F*, μN		10.5 ± 0.5	10.5 ± 0.5	18.3 ± 1.0	14.4 ± 1.3

**Table 3:** Elemental compositions of the surface layer of initial and He^+^-irradiated “MWCNT/Ti” systems according to XPS data.

Sample	Concentration, at. %		
	[C]	[O]	[Ti]
Initial MWCNT/Ti	93.80	5.21	0.99
MWCNT/Ti, irradiated for 10 min	85.88	13.38	0.74
MWCNT/Ti, irradiated for 20 min	83.01	14.93	2.06
MWCNT/Ti, irradiated for 30 min	80.38	16.45	3.17

**Table 4:** The results of a three-component approximation of the intensity distribution in the O 1s PE spectra and an estimation of the concentrations of various oxygen-containing components for the initial and He+-irradiated “MWCNT/Ti” systems.

Sample	Relative Component Intensity, %			Concentration, at.%			
	O1	O2	O3	O_tot_	O1	O2	O3
Initial MWCNT/Ti	6.7	79.6	13.7	5.21	0.35	4.14	0.72
MWCNT/Ti, irradiated for 10 min	19.6	56.5	23.9	13.38	2.62	7.56	3.20
MWCNT/Ti, irradiated for 20 min	19.8	51.8	28.4	14.93	2.95	7.74	4.24
MWCNT/Ti, irradiated for 30 min	27.8	46.5	25.7	16.45	4.57	7.65	4.23

## Data Availability

Data are contained within the article.
